# Long Follow-Up Times Weaken Observational Diet–Cancer Study Outcomes: Evidence from Studies of Meat and Cancer Risk

**DOI:** 10.3390/nu16010026

**Published:** 2023-12-21

**Authors:** William B. Grant

**Affiliations:** Sunlight, Nutrition, and Cancer Research Center, P.O. Box 641603, San Francisco, CA 94164-1603, USA; wbgrant@infionline.net

**Keywords:** bladder cancer, breast cancer, case–control, cohort, colorectal cancer, ecological, follow-up time, gastric cancer, 25-hydroxyvitamin D, vitamin D

## Abstract

For years, prospective cohort studies of diet and cancer incidence have reported smaller effects than do retrospective case–control (CC) studies. The differences have been attributed to problems with CC studies, including dietary recall bias, poor matching of cases and controls, and confounding. The hypothesis evaluated here is that long follow-up periods between ascertainment of diet and cancer incidence weaken the findings. Prospective studies of cancer incidence with respect to serum 25-hydroxyvitamin D concentration have already shown reduced benefit of higher concentrations for longer follow-up periods. Evaluating that hypothesis for dietary factors involved searching the journal literature for meta-analyses of red meat and processed meat and cancer incidence. I used findings from observational studies for bladder, breast, colorectal, and gastric cancers. To evaluate the effect of duration of follow-up time, I used two approaches. First, I plotted the relative risks for CC studies for gastric cancer with respect to consumption of 100 g/day of red meat and for bladder cancer for 50 g/day of processed meat against the interval between the dietary data and cancer incidence. Second, I compared nested CC studies of meat and cancer incidence for five breast cancer studies and one colorectal cancer study. Both approaches yielded an inverse correlation between interval or follow-up time and relative risk. My findings strongly suggest that diet near time of cancer diagnosis is more important than for longer intervals, that results from meta-analyses should be revised when possible with appropriate adjustments for duration of follow-up, and that dietary guidelines be revised accordingly.

## 1. Introduction

A 2006 review of nutritional epidemiology included observational studies, such as ecological correlation, migrant studies, and time-trend studies; analytical studies, such as retrospective case–control (CC), hospital-based controls, population-based controls, prospective cohort, and nested CC studies; and intervention studies [1]. CC and prospective cohort studies are the most common type and are the subject of this review.

CC studies have advantages in that they require fewer participants and no follow-up [1]. Drawbacks generally include recall bias and selection bias. The main advantage of prospective cohort studies is that dietary exposures are collected before disease develops. A caveat is that repeated assessments of diet should be obtained to add dietary factors and to study major dietary changes. However, as discussed later, repeated assessments of diet are seldom done in such studies; if they are, it is generally every 4 years. The authors noted that prospective cohort studies have reported weaker or null associations between diet and cancer than those from CC studies. A proposed explanation was that cancer patients may have erroneously recalled their prediagnosis diet because they had a preconception regarding how diet might influence disease or their current diet changed because of the diagnosis. Also, prospective cohort studies might have included more confounders.

**Figure 1:**
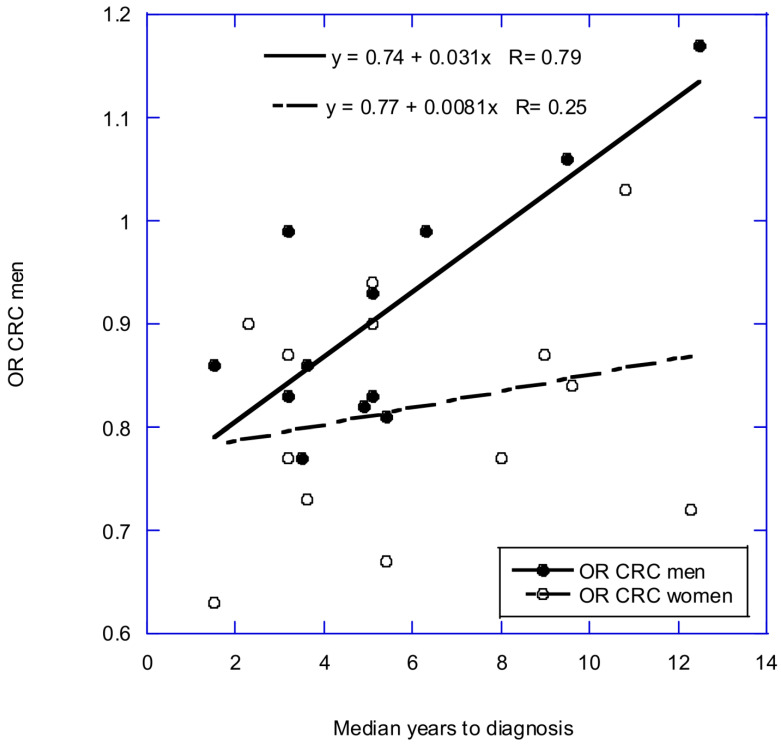
Effect of follow-up time for colorectal cancer incidence with respect to serum 25(OH)D concentration at baseline. Used with permission from Muñoz and Grant [4]. CRC, colorectal cancer; OR, odds ratio.

The follow-up period can affect the outcome of observational studies related to serum 25-hydroxyvitamin D [25(OH)D] in relation to cancer [2,3,4] and mortality rates [5]. In support, a 25(OH)D tracking study showed that the correlation coefficient between 25(OH)D measurements from 1994 and 2008 ranged from 0.42 to 0.52 [6]. Here is an example regarding serum 25(OH)D concentration and incidence of colorectal cancer (CRC) based on data in a 2019 article [7] published in a 2022 review [4]. The 2019 article reported that for each 10 ng/mL increment in circulating 25(OH)D, CRC risk was 19% lower in women (relative risk [RR] = 0.81 [95% confidence interval = 0.75–0.87]) and 7% lower in men (RR = 0.93 [95% CI = 0.86–1.00]). As shown in Figure 1, the reduction is nearly the same for both men and women after consideration of follow-up time.

Good evidence also indicates that dietary intake changes with time. For example, from 1999–2000 through 2017–March 2020, US obesity prevalence increased from 30.5% to 41.9%. During the same time, the prevalence of severe obesity increased from 4.7% to 9.2% [8]. That is an increase of 1.8% per year compounded, resulting in a 19% increase in 10 years.

Global meat consumption patterns have changed since 1961. A 2015 review reported that meat consumption is highly correlated with annual per capita gross domestic product [9]. Increases between 1961 and 2011 were particularly high in Brazil, China, Japan, and Spain.

This article strives to determine whether CC or prospective cohort studies have yielded the most accurate determination of diet’s role in cancer risk. For that purpose, I examine CC and prospective cohort studies mostly after 2000 regarding the correlations of red and processed meat with incidence of bladder, breast, CRC, and gastric cancer. I also compare the results with those from a recent prospective cohort study regarding food groups and risk of chronic diseases in which dietary intake was assessed every 4 years [10].

## 2. Materials and Methods

The hypothesis to be tested is that longer follow-up periods in observational studies of diet and cancer risk weaken their outcomes. I use results from red and processed meat. For CC and prospective cohort studies, the starting point was to find meta-analyses through Google Scholar and the National Library of Medicine’s PubMed database. Search terms included “case–control”, “cohort”, “prospective”, “nested case–control”, “meat”, “red meat”, “processed meat”, “meta-analysis”, “ecological”, and “cancer”. In the first literature search, I sought suitable observational studies of cancer risk with respect to red meat and processed meat to evaluate the hypothesis that as the interval between the dietary data and cancer diagnosis increased, the apparent risk decreased. I also sought ecological studies that reported cancer risk with respect to meat in either countries or groups of people from different countries living in one country. The goal of that effort was to determine whether more evidence existed that meat increased cancer risk.

The second part of the study examined the effect on relative risk for red and processed meat in CC studies on the basis of consuming 100 g/day for red meat with respect to the interval between the dietary data and cancer diagnosis. All studies were scrutinized for reports of RRs and their 95% confidence intervals according to quantity of meat intake. These were abstracted and placed into tables. If RRs were not provided in the published report, the data were not included in the analysis.

The third part of this study examined the effect of the interval between dietary data collection and end of study in nested case–control (NCC) studies. This analysis examines breast cancer because it had the most NCC studies of red meat and cancer risk.

## 3. Results

### 3.1. Data Used in This Article

The meta-analyses used in this work are listed in Table 1, Table 2, Table 3 and Table 4. Those datasets were determined to be most appropriate for use according to number of studies included in the meta-analysis being in general agreement with other meta-analyses for the same type of cancer, and being more recent. Results for CC studies clearly indicate higher risk of cancer than do cohort studies.

The hypothesis considered here is that the longer the follow-up time in an observational study, the lower will be the apparent RR. I tried to examine that idea by using cohort studies, but no effect of follow-up time was evident. Follow-up times in cohort studies generally run from 4 to about 20 years. Thus, the idea arose to investigate this hypothesis by using data from CC studies. (I agree with Barnard, Willett, and Ding [17] that the most important contribution of meta-analysis is not necessarily the single statistical summary of the size effect but rather the ability to elucidate why different studies have produced different results). The first effort was with respect to RRs comparing high and low consumption rates. Unfortunately, the range of dietary intake has too much variability for that approach to work well. The next idea was to use CC data for 50 or 100 g of meat/day for various cancers. Gastric and bladder cancers had many such CC studies, so I chose them for this evaluation. The data used for gastric and bladder cancer are given in Table 5 and Table 6, respectively. The tables exclude studies for which the interval between diagnosis and dietary data could not be identified. Also, a gastric cancer study from New Zealand involving Maori was excluded since it was on people in a culture with a much different diet than in the other studies [18].

**Figure 2:**
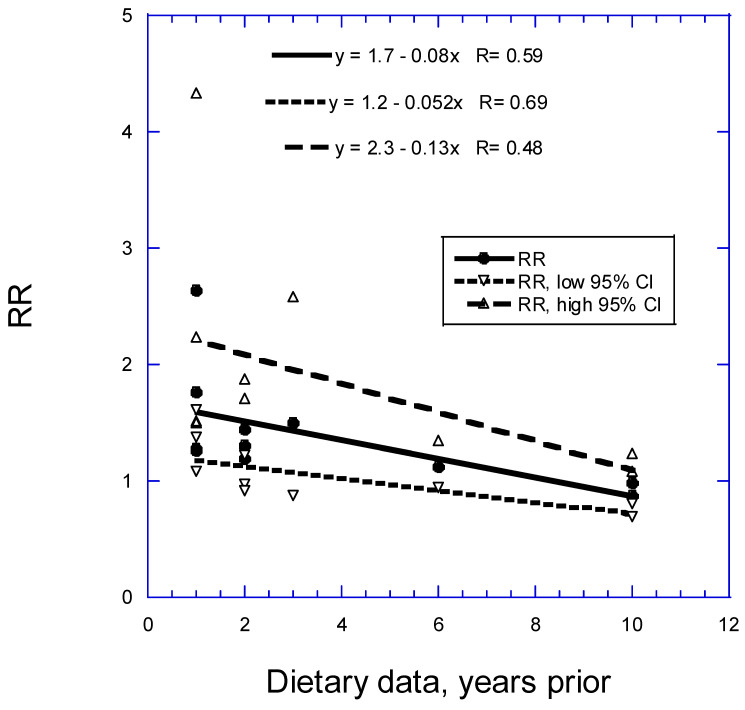
Relative risk of gastric cancer with respect to 100 g/day of red meat from case–control studies with different periods for dietary assessment. 95% CI, 95% confidence interval; CC, case–control; RR, relative risk.

Figure 2 shows that the RR in CC studies for gastric cancer incidence related to 100 g/day of red meat goes from 1.6 (95% CI, 1.3–2.4) at 1 year to 0.9 (95% CI, 0.8–1.2) at 10 years, i.e., is reduced by 0.08 per added year of follow-up and is no longer significant beyond about 4 years.

**Figure 3:**
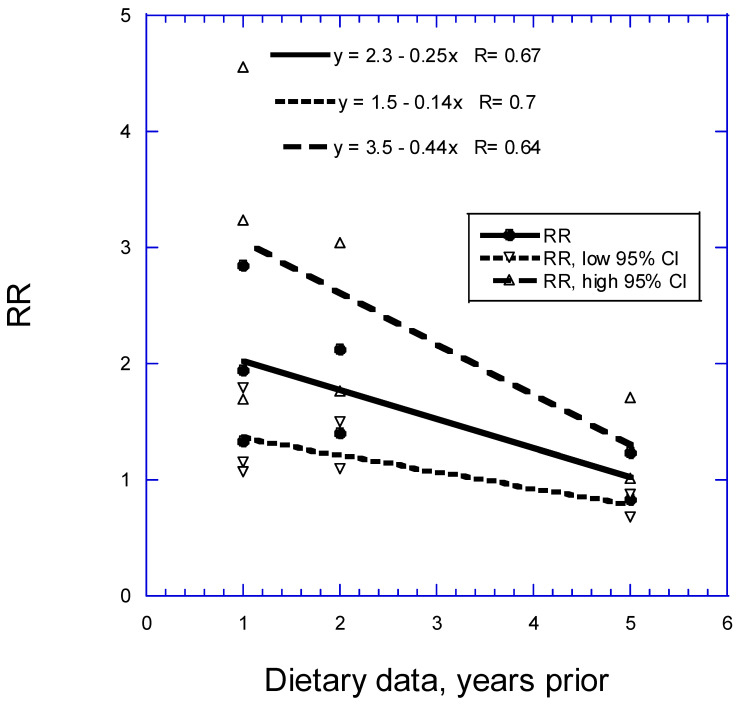
Relative risk of bladder cancer with respect to 100 g/day of red meat from case–control studies with different periods for dietary assessment. 95% CI, 95% confidence interval; CC, case–control; RR, relative risk.

Figure 3 shows that the RR for bladder cancer with respect to 100 g/day of red meat goes from 2.1 (95% CI, 1.4–3.1) for 1 year of follow-up to 1.1 (95% CI, 0.8–1.3) for 5 years of follow-up, i.e., is reduced by 0.3 per added year of follow-up, and is no longer significant after about 4 years.

### 3.2. Recall Bias

An alternate hypothesis for higher inverse correlations between dietary factors and risk of cancer is recall bias. As seen in Table 5 and Table 6, participants in CC studies were asked to recall dietary intake 5–10 years before cancer diagnosis in the earlier studies. That practice gave rise to the development of the recall bias hypothesis, i.e., that cancer patients were more likely than controls to know which foods were risk factors for cancer and thus change their responses accordingly. However, as shown in Figure 2 and Figure 3, the highest RRs were for studies with the shortest follow-up times. Thus, recall bias cannot explain that CC studies have higher RRs than prospective cohort studies.

A 2005 review stated that CC studies of diet and cancer are plagued by recall bias, selection of control bias, and confounding [33]. That review also suggested that self-reported dietary information may be less reliable than that obtained by an interview. The author also suggested that randomized controlled trials might be a way to determine diet’s effect on cancer risk, though he admitted that approach would be difficult.

My opinion is that recall of dietary components and amounts decreases with time. Dietary changes between the period of interest and the time of recall also will decrease reliability of recall. Participant-completed dietary questionnaires probably yield less reliable data than can be obtained by interviews. Confounding by various dietary factors also can adversely affect interpretation of results. However, dietary changes between older periods and time of diagnosis can also reduce the observed RR. That reasoning prompts the question of how to differentiate between recall bias and dietary change.

Another way to evaluate the effect of follow-up time is to use NCC studies. NCC studies are a hybrid of cohort and CC studies in which participants in cohort studies who develop the disease of interest, such as cancer, are matched to other participants who did not. Dietary data are obtained at enrollment and, in some studies, at later times. NCC studies use dietary data obtained at enrollment, and cohort participants who develop cancer are compared with suitable controls. Through Google Scholar, I found NCC studies of meat and cancer risk—the most common results being for breast cancer. Table 7 gives the characteristics and RR findings for breast cancer NCCs with mean follow-up times of 2–10 years. The *p* values for trends are included if reported in the articles. What is clear is that only NCC studies with follow-up times less than 5 years reported significant meat-related risk. Thus, those findings support this article’s hypothesis that long follow-up times reduce observational studies’ ability to find significant associations with dietary factors.

An important observational study regarding optimal dietary patterns for prevention of chronic disease was published in 2023 [10]. It was based on data from three cohorts: 162,667 women from the Nurses’ Health Study and Nurses’ Health Study II as well as 43,185 men from the Health Professionals Follow-up Study, all run by Harvard University, monitored for up to 32 years. An important factor was that diet was assessed every 4 years. The study assessed the contribution of 37 categories of food for risk of disease and developed both a reversed empirical dietary index for hyperinsulinemia and a reversed empirical dietary inflammatory pattern. Red and processed meat had the highest negative effects on the various health outcomes considered, including cancer. That article played an important role in preparing my review of diet’s role in risk of Alzheimer’s disease (AD) [40].

### 3.3. How Red Meat and Processed Meat Increase Cancer Risk

Red and processed meat increase cancer risk through several mechanisms. A large cohort study with a 7-year follow-up period reported these components of red or processed meat as associated with CRC incidence: The potential mechanisms for this relation include heme iron, nitrate from processed meats, and heterocyclic amine intake [41]. A 2010 review added fatty acid content, noting that red meat had as high as 30–40% fat, with saturated fat’s accounting for 40–50% of the fat in lean meat [42]. The review also noted that poultry has lower saturated fat content. That finding may explain why white meat (chicken, duck, turkey, and rabbit) had a lower risk for gastric cancer than red or processed meat, as found by Kim and colleagues [11], included in Table 1.

A 2017 review of the thermal effects in preparing red meat discussed compounds and their mechanisms, including polycyclic aromatic hydrocarbons, heterocyclic amines, N-nitroso compounds, heme iron, and macromolecular oxidation products. Their carcinogenic mechanisms were then addressed with further emphasis on the involvement of inflammation and oxidative stress [43].

A 2018 review discusses the role of heterocyclic amines and polycyclic aromatic hydrocarbons in carcinogenesis induced by DNA mutation [44]. The review also discusses that heme, through the lipid peroxidation process and therefore formation of N-nitroso compounds, produces cytotoxic and genomic aldehydes. It also reviews a recent hypothesis regarding the combined actions between N-glycolylneuraminic acid and genotoxic compounds [45].

The beef interests naturally dispute the evidence regarding heme in red meat as an important risk factor for colon cancer [46]. In the online International Committee of Medical Journal Editors disclosure of potential conflicts of interest, both authors disclosed having received consulting fees from the National Cattlemen’s Beef Association.

Eating red and processed meat also is an important risk factor for obesity. A 2014 systematic review (*N*  =  1,135,661) and a meta-analysis (*N*  =  113,477) of observational studies were conducted with 21 and 18 studies, respectively. The meta-analysis showed that consuming more red and processed meats was a risk factor for obesity (OR = 1.37 [95% CI, 1.14–1.64]) [47]. Obesity increases cancer risk through a combination of increasing systemic inflammation and risk of hyperinsulinemia [48].

### 3.4. Ecological Studies

Ecological studies based on dietary supply data for several countries or trends in single countries are useful for comparison with retrospective CC and prospective observational studies.

Meat’s role in cancer risk was a hot topic shortly after 1900. A *New York Times* story from 24 September 1907 reported the findings of Dr. G. Cooke Adams, whom the Chicago Department of Health had hired to study Chicago’s increasing cancer burden [49]. The first 7 years of the century saw 178 deaths from cancer among 58,835 Chicago-born residents but 4463 cancer deaths among 61,019 deaths of foreign-born inhabitants. People born in Germany, Ireland, Scandinavia, and the Slavic countries had much higher cancer death rates than residents of their native countries. The Italians and Chinese had low cancer death rates in Chicago. The Italians ate macaroni and spaghetti, whereas the Chinese ate rice. The high rates of cancer deaths among the northern and Eastern Europeans were attributed to increased consumption of animal foods, particularly those derived from diseased animals. Slaughtered livestock in those days had a high prevalence of tuberculosis, actinomycosis, and cancer. However, I believe the culprit was the meat itself, not the diseases.

A 1910 book elaborated on cancer rates in 1900 by countries [50]. In 1900, cancer death rates (per 100,000/year) for mothers of white populations in the US from various countries included 25 from Italy, 29 from Russia and Poland, 55 from Scandinavia, 53 from the US, 78 from England and Wales, 84 from Ireland and Germany, 90 from Scotland, and 98 from France. The low rates for people from Russia and Poland were attributed to their being Jewish and having an aversion to meat. Higher rates among immigrants from England, Germany, and Ireland were attributed to their ancestors’ being in the US or Canada for one or two generations, thereby achieving greater prosperity, which allowed them to purchase meat.

The best known multicounty ecological study is the 1975 study by Armstrong and Doll [51]. They used dietary supply data for incidence rates for 27 cancers in 23 countries. The cancer incidence data were from the International Union Against Cancer between 1966 and 1970 [52,53]. The age range was 35–64 years. Dietary supply data were obtained from the UN’s Food and Agriculture Organization from 1959, 1960, and 1971 and from other sources up to 1972. Meat was highly correlated with gross domestic product, sugar, eggs, and fish, and was strongly inversely correlated with cereals and pulses. For colon and rectal cancer incidence, meat had the highest correlation, *r* = 0.68 to 0.89, respectively. For breast and endometrial cancer, meat, with *r* = 0.78, was slightly behind total fat (*r* = 0.79 and 0.85, respectively) and gross national product (*r* = 0.83 and 0.82, respectively). For renal cancer, *r* = 0.70 for men and 0.73 for women, which was comparable to *r* for gross national product, animal protein, and total fat. Of course, meat is a major component of animal protein and total fat.

A 2006 ecological study compared meat consumption and the polymorphic gene *NAT2* with CRC incidence in 27 countries [54]. The cancer data came from GLOBOCAN for 1988–1992 [55]. The food supply data were from FAOSTAT 2004 [56]. The *NAT2* data were from Brockton and colleagues [57]. Meat had the highest unadjusted correlation coefficient of 0.82 for both males and females, rising to 0.86 as a partial correlation coefficient when adjusted for *NAT2* and slightly lower values when adjusted for other factors including fish, animal fat, vegetables, fruit, and alcohol. Models were developed starting with meat alone, which explained 70% (*p* < 0.0001) of the intercountry variance for males and 69% (*p* < 0.0001) for females. Adding *NAT2*, vegetables, and fish resulted in explaining 86% (*p* = 0.01) of the variance for males. The same without fish resulted in explaining 81% (*p* = 0.02) of the variance for females.

Ecological studies are considered hypothesis generating [1]. However, in my experience, they can be accurate. As an aside, the Armstrong and Doll study led to my study of diet and prevalence of AD in 11 countries [58], and solar UVB/vitamin D and cancer mortality rates in the US [59]. The AD study showed total fat and total energy (caloric supply) highly correlated with AD; a recent update reported that meat had a slightly higher correlation with AD than fat and energy [40]. A later UVB/vitamin D and cancer study [60] included consideration of confounding factors with the finding that the correlations with solar UVB doses were little changed. The results are well supported by other studies for cancer [4]. Another ecological study of dietary meat and expression of rheumatoid arthritis [61]—noting that significant adverse effects of rheumatoid arthritis increased significantly after 1970 in many European countries after meat consumption increased—has been well supported by later studies that removed meat from the diet [62,63].

## 4. Discussion

This study showed in two ways that the longer the follow-up period, the lower the apparent RR for cancer with respect to red and processed meat. For follow-up periods of 10 years or longer, practically no apparent risk is involved. The proposed explanation is that cancer risk factor values change with time. That could be the amount of meat consumed but could also be several other cancer risk–modifying factors, including other dietary components, body mass index, and serum 25(OH)D concentration. Global and US obesity rates have increased rapidly in the past two to three decades [64,65]. Serum 25(OH)D concentrations significantly affect risk of most cancers [4]. Meat is an important source of vitamin D in the form of 25(OH)D. A UK study involving white residents showed that meat eaters had higher 25(OH)D concentrations than fish eaters, with values about 8 ng/mL higher than those of vegans [66]. Apparently the amount of 25(OH)D in meat does not offset its increased cancer risk.

The most likely explanation for the decreasing relationship between dietary factors and cancer incidence with increasing time interval is that diet changes over time. The findings regarding time interval for serum 25(OH)D seem to be very well explained by changes over time for cancer incidence [4] and all-cause mortality rate [5]. Cancer risk increases with age especially above the age of 60 years. People often change lifestyle around retirement age such as by moving, exercising less, gaining weight, etc. It is also noted that obesity rates have increased significantly in the past one-to-two decades, with consumption of ultra-processed foods playing an important role. Higher adiposity is a risk factor for cancer through increase in systemic inflammation by production of different pro-inflammatory cytokines [67].

There are three stages of cancer, initiation, promotion, and progression. Angiogenesis is important for promotion and metastasis is important for progression. Vitamin D reduces cancer initiation through effects on cells, but also reduces angiogenesis around tumors and metastasis into surrounding tissues [4]. Interestingly, the effect of vitamin D on cancer mortality rate is stronger than its effect on cancer incidence, e.g., [4,68]. The difference is likely due to the fact that there are many mechanisms related to cancer incidence but few natural mechanisms against angiogenesis or metastasis. Immunosenescence helps explain why cancer incidence rates increase with age [69]. However, immunosenescence seems to have a lesser role in tumor progression.

Assuming that diet near time of cancer incidence is most important, the findings from retrospective CC studies with short times between dietary assessment and diagnosis should be used to determine how diet affects cancer risk. Doing so will result in improved guidelines for preventing cancer in general. If the same factors also affect cancer survival, then people diagnosed with cancer can change their diet and vitamin D status to improve survival odds. For example, vitamin D supplementation of 2000 IU/day for digestive tract cancer patients in the p53-immunoreactive subgroup had about a 70% lower risk of death during 7 years than those given a placebo [70]. A German observational study involving 104 long-term CRC survivors (median: 6 years) reported that higher healthful plant-based diet scores showed a strong tendency toward lower mortality (HR, 0.82 [95% CI, 0.67–1.01]). The unhealthful plant-based diet index was associated with higher mortality but lost statistical significance after multivariable adjustment (HR, 1.19 [95% CI, 0.96–1.48]) [71]. The Women’s Health Initiative Dietary Modification clinical trial included 48,835 postmenopausal women [72]. Those who adopted a low-fat dietary pattern associated with increased vegetable, fruit, and grain intake had a 21% reduction in breast cancer deaths (HR = 0.79 [95% CI, 0.64–0.97]). A 2022 systematic review and meta-analysis on diet’s role in prognosis among cancer survivors included 35 prospective cohort studies and 14 randomized controlled trials published between 2011 and 2021 [73]. Better overall diet quality was associated with improved overall survival among breast and CRC survivors; adherence to the Mediterranean diet was associated with lower risk of mortality in survivors of CRC and prostate cancer.

It is instructive to look at dietary guidelines regarding meat. A 2019 global review included statistics on the percentage of county food-based dietary guidelines regarding meat [74]. Among the key messages, 34% recommended choosing lean meats or removing fat from meat, 13% recommended limiting or moderating meat consumption in general, and 11% recommended limiting or moderating consumption of specific types of meat (red, processed, and/or cured).

The 2019 EAT-*Lancet* Commission on healthy diets from sustainable food systems included this key message: “Transformation to healthy diets by 2050 will require substantial dietary shifts, including a greater than 50% reduction in global consumption of unhealthy foods, such as red meat and sugar, and a greater than 100% increase in consumption of healthy foods, such as nuts, fruits, vegetables, and legumes. However, the changes needed differ greatly by region” [75].

A 2018–2019 survey of meat-reduced dietary practices in Australia, Canada, Mexico, the UK, and the US reported about 40% had made efforts in the past year to reduce consumption of any meat, about 35% for red meat, and about 25% same for consuming less of all meats [76].

A 2020 review by researchers who favor meat consumption noted that mainstream dietary recommendations now commonly advise people to minimize intake of red meat for health and environmental reasons. Most recently, a major report from the EAT-*Lancet* Commission recommended a planetary reference diet based mostly on plants and with no or very low (14 g/day) consumption of red meat. The abstract states that “We argue that claims about the health dangers of red meat are not only improbable in the light of our evolutionary history, they are far from being supported by robust scientific evidence” [77]. The lead author is a member of such organizations as the Belgian Association of Meat Science and Technology.

A 2023 review led by the same author stated that “Meat supplies high-quality protein and various nutrients, some of which are not always easily obtained with meat-free diets and are often already suboptimal or deficient in global populations. Removal of meat comes with implications for a broad spectrum of nutrients that need to be accounted for, whereas compensatory dietary strategies must factor in physiological and practical constraints” [78]. In particular, the authors noted a few nutrients that meat has, such as vitamin B_12_, trace minerals, and bioactive compounds with health-improving potential, and would be particularly helpful to improve nutritional status in regions in the Global South that rely heavily on cereals. The authors write as though they receive funding from meat interests.

One problem with accepting cohort studies of meat as correct and CC studies and ecological studies as incorrect is that health policy decisions are made using results of cohort studies. That was done recently. A 2019 review by 18 members from the Nutritional Recommendations Consortium stated: “The panel suggests that adults continue current unprocessed red meat consumption (weak recommendation, low-certainty evidence). Similarly, the panel suggests adults continue current processed meat consumption (weak recommendation, low-certainty evidence). Our weak recommendation that people continue their current meat consumption highlights both the uncertainty associated with possible harmful effects and the very small magnitude of effect, even if the best estimates represent true causation, which we believe to be implausible” [79]. A correction to that review noted that lead author Bradley Johnston did not initially indicate a grant from Texas A&M AgriLife Research to fund investigator-driven research related to saturated and polyunsaturated fats.

A recent review used cohort studies from global, Asian, European, and North American studies to evaluate the health risks of noncommunicable disease risk associated with red and processed meat consumption by some of the above authors [80]. They summarized their findings based on cohort studies as thus: “While some researchers claim that red meat consumption is intrinsically harmful, the evidence does not support this being the case where intakes are below 75 and 20 g/d, respectively. Even beyond these intake levels, only small increases in relative risks are reported (<25%), there is little to no effect on absolute risk, and the certainty of evidence remains low to very low based on the best available summary evidence”.

## 5. Conclusions

The findings in this article suggest several actions:

1. In terms of new observational studies of diet and risk of disease, dietary intake should be assessed within 4 years before diagnosis, with shorter times preferred. Earlier times of diet assessment appear less likely to reveal associations but may also be included when available.
2. CC or NCC studies should be preferred over cohort studies whenever possible, reducing time and effort needed for collecting data and conserving biological specimens.
3. Observational studies with short follow-up times or intervals between disease diagnosis and dietary data should be given equal or higher standing than those with longer times in assessing diet’s role in risk of disease.
4. Previous meta-analyses of both CC and cohort studies of dietary intake and disease outcomes should be revised when possible, with appropriate adjustments for interval or duration of follow-up.

## Figures and Tables

**Table 1:** Data from meta-analyses for gastric cancer.

Study	Meat	Comparison	*N*	Yrs	RR (95% CI)	Ref.
CC, pop	Red	High vs. low	12	1997–2014	1.42 (1.12–1.82)	[11]
CC, hosp	Red	High vs. low	8	1997–2012	1.81 (1.41–2.33)	
CC	Red	Per 100 g/day	14	1997–2017	1.31 (1.13–1.42)	
CC, pop	Processed	High vs. low	11	1990–2012	1.58 (1.32–1.89)	
CC, hosp	Processed	High vs. low	12	1997–2012	2.03 1.56–2.68)	
CC	Processed	Per 50 g/day	12	1990–2014	2.17 (1.51–3.11)	
CC, pop	White	High vs. low	9	1998–2013	0.75 (0.61–0.93)	
CC, hosp	White	High vs. low	8	2001–2011	0.81 (0.61–1.06)	
CC	White	Per 100 g/day	10	1998–2011	0.65 (0.35–1.25)	
Cohort	Red	High vs. low	6	2005–2020	1.09 (0.94–1.26)	[12]
Cohort	Processed	High vs. low	10	1990–2020	1.15 (0.96–1.37)	

95% CI, 95% confidence interval; CC, case–control; hosp, hospital based; *N*, number of studies; pop, population based; RR, relative risk.

**Table 2:** Data from meta-analyses for colorectal cancer.

Study	Meat	Comparison	*N*	Yrs	RR (95% CI)	Ref.
CC	Red	High vs. low	14	1984–1999	1.36 (1.17–1.59)	[13]
CC	Processed	High vs. low	16	1973–1999	1.29 (1.09–1.52)	
Cohort	Red	High vs. low	22	1997–2020	1.10 (1.03–1.17)	[12]
Cohort	Processed	High vs. low	23	1997–2020	1.18 (1.13–1.24)	

95% CI, 95% confidence interval; CC, case–control; *N*, number of studies; RR, relative risk.

**Table 3:** Data from meta-analyses for breast cancer.

Study	Meat	Comparison	*N*	Yrs	RR (95% CI)	Ref.
CC	Red	High vs. low	9	1991–2015	1.55 (1.26–1.91)	[14]
Cohort	Total red	High vs. low	16	1989–2020	1.09 (1.03–1.15)	[12]
Cohort	Processed	High vs. low	16	1999–2020	1.06 (1.01–1.12)	

95% CI, 95% confidence interval; CC, case–control; *N*, number of studies; RR, relative risk.

**Table 4:** Data from meta-analyses for bladder cancer.

Study	Meat	Comparison	*N*	Yrs	RR (95% CI)	Ref.
CC	Red	High vs. low	9	1991–2012	1.23 (0.91–1.67)	[15]
CC	Processed	High vs. low	6	1991–2012	1.46 (1.10–1.95)	
CC	Red	Per 100 g/day	7	2000–2011	1.94 (1.16–3.24)	[16]
CC	Processed	Per 50 g/day	6	2007–2014	1.31 (1.06–1.63)	
Cohort	Red	High vs. low	5	2000–2011	1.08 (0.97–1.20)	[15]
Cohort	Processed	High vs. low	5	2000–2011	1.08 (0.96–1.20)	
Cohort	Red	Per 100 g/day	6	2000–2013	1.01 (0.97–1.06)	[16]
Cohort	Processed	Per 50 g/day	5	2000–2010	1.10 (0.95–1.27)	

95% CI, 95% confidence interval; CC, case–control; *N*, number of studies; RR, relative risk.

**Table 5:** Data for gastric cancer in case–control studies, 100 g/day of red meat from Kim and colleagues [11].

N	Yr Published	Yrs before Dietary Data	RR (95% CI)	Ref.
154	1997	1	1.28 (1.08–1.52)	[19]
770 M	1998	10	0.99 (0.80–1.23)	[20]
354 F	1998	10	0.87 (0.70–1.09)	[20]
745	2000	2	1.45 (1.22–1.71)	[21]
274	2004	6	1.13 (0.95–1.35)	[22]
1180	2008	2	1.19 (0.97–1.46)	[23]
217	2009	1	2.64 (1.61–4.34)	[24]
275	2009	1	1.27 (1.08–1.50)	[25]
128	2009	1	1.76 (1.38–2.24)	[26]
230	2013	2	1.31 (0.92–1.87)	[27]
226	2014	3	1.50 (0.87–2.58)	[28]

95% CI, 95% confidence interval; F, females; M, males; *N*, number of cases; RR, relative risk.

**Table 6:** Data for bladder cancer relative risk in case–control studies for 100 g/day of red meat from Crippa and colleagues [16].

N	Yr Published	Yrs before Dietary Data	RR (95% CI)	Ref.
450	2000	2	2.13 (1.50–3.04)	[21]
912	2007	5	0.84 (0.68–1.02)	[29]
1029	2008	2	1.40 (1.10–1.77)	[23]
254	2009	1	1.34 (1.07–1.69)	[25]
884	2012	1	2.85 (1.79–4.55)	[30]
1000	2012	5	1.23 (0.88–1.71)	[31]
500	2012	1	1.94 (1.16–3.24)	[32]

95% CI, 95% confidence interval; HR, hazard ratio; *N*, number of cases.

**Table 7:** Nested case–control studies for breast cancer and CRC.

Cancer	Population	Mean Follow-Up	Meat Type	Doneness, RR (95% CI)	Ref.
Breast	Iowa, USA *N*_c_ = 227; *N*_co_ = 603	2 years	Hamburger	R–M; 1.0 WD; 1.23 (0.89–1.71) VWD; 1.54 (0.96–2.47) *p* = 0.04	[34]
	Iowa, USA *N*_c_ = 249; *N*_co_ = 598	2 years	Beefsteak	R–M; 1.0 WD; 1.22 (0.89–1.71} VWD; 2.21 (1.30–3.77) *p* = 0.04	
	Iowa, USA *N*_c_ = 260; *N*_co_ = 436	2 years	Bacon	R–M; 1.0 WD; 1.26 (0.71–2.22) VWD; 1.64 (0.92–2.91) *p* = 0.01	
				g/day, RR (95% CI)	
Breast	New York *N*_c_ = 180; *N*_co_ = 180	3 years	Total meat	8 g/day; 1.0 20; 1.11 (0.63–2.02) 30; 1.88 (1.10–3.21) 44; 1.62 (0.93–2.82) 73; 1.87 (1.09–3.21) *p* = 0.01	[35]
Breast	The Netherlands *N*_c_ = 229; *N*_co_ = 263	3.8 years 22 ± 18 months *	Fresh red	<30: 1.00 30–44: 1.31 (0.83–2.05) >45: 1.30 (0.83–2.02)	[36]
	*N*_c_ = 229; *N*_co_ = 262		Processed	<20; 1.00 20–34; 0.95 (0.61–1.49) >35; 1.05 (0.67–1.64)	
Breast	USA *N*_c_ = 455; *N*_co_ = 462	5 years	Red, incl. fresh and processed	≤0.5 s/day; 1.0 0.51–1.0 s/day; 0.9 (0.7–1.3) >1.0 s/day; 0.9 (0.6–1.3)	[37]
	USA *N*_c_ = 455; *N*_co_ = 462		Processed	≤0.14 s/day; 1.0 0.15–0.50 s/day; 1.3 (1.0–1.8) >0.50 s/day; 1.0 (0.7–1.5)	
CRC	CA, HI, USA *N*_c_ = 1009; *N*_co_ = 1522	5 years	Red	<10.4 g/1000 kcal/day; 1.0 10.4–<17.7; 1.11 (0.80–1.28) 17.7–<26.0; 0.96 (0.74–1.23) *p* = 0.67	[38]
	*N*_c_ = 1009; *N*_co_ = 1522		Processed	<3.54 g/1000 kcal/day; 1.0 3.5–<6.7; 1.04 (0.82–1.32) 6.7–<11.0; 1.13 (0.89–1.44) ≥11.0; 1.08 (0.8–1.39) *p* = 0.46	
Breast	Milan, Italy *N*_c_ = 3156; *N*_co_ = 9413	10 years	Red	<1 s/wk; 1.00 1 s/wk; 1.01 (0.90–1.12) 2–3 s/wk; 0.97 (0.87–1.08) ≥4 s/wk; 1.12 (0.96–1.31) *p* = 0.58	[39]
	Milan, Italy *N*_c_ = 3165; *N*_co_ = 9503	10 years	White	<1/wk; 1.00 1/wk; 1.06 (0.93–1.22) 2–3/wk; 1.14 (1.00–1.30) ≥4/wk; 1.09 (0.92–1.28) *p* = 0.11	

* median time between enrollment and diagnosis; 95% CI, 95% confidence interval; CRC, colorectal cancer; *N*_c_, number of cases; *N*_co_, number of controls; R–M, rare to medium; RR, relative risk; s/day, servings/day; s/wk, servings/week; VWD, very well done; WD, well done.

## Data Availability

All data and material are included in the manuscript.
